# Research on the Decoupling of Agricultural Planting Carbon Intensity and Food Security in Hunan Province, China

**DOI:** 10.3390/foods15101635

**Published:** 2026-05-08

**Authors:** Yue Xing, Xianzhao Liu, Hai Xiao, Chenxi Dou

**Affiliations:** 1 School of Earth Science and Space Information Engineering, Hunan University of Science and Technology, Xiangtan 411201, China; 2 Hunan Engineering Research Center of Natural Ecosystems Carbon Sink Monitoring, Changsha 410000, China

**Keywords:** agricultural planting, carbon emission intensity, food security, Tapio decoupling model

## Abstract

Faced with the dual challenges of intensifying global climate change and tightening food security, achieving a balance between food security and agricultural carbon sequestration and emissions reduction has become a focal point of academic inquiry. This study quantifies agricultural carbon intensity and food security levels in Hunan Province from 2002 to 2023. By employing the Tapio decoupling model, the Logarithmic Mean Divisa Index (LMDI) method, and spatial analysis techniques, it systematically examines the decoupling relationship and driving mechanisms between agricultural carbon intensity and food security in Hunan Province. The results indicate that agricultural carbon intensity exhibits a spatial pattern of “high in the east, low in the west,” while food security levels decline from the eastern plains to the western mountainous regions. The decoupling trajectory is broadly characterized by a transition from predominantly weak decoupling toward strong decoupling; since 2016, prefecture-level cities exhibiting strong decoupling have accounted for 92.9% of all cases, displaying spatial characteristics of “overall improvement, an uneven process, and regional asynchrony.” Agricultural energy intensity, energy structure, and rural labor force size serve as positive drivers of decoupling between agricultural carbon intensity and food security, whereas agricultural economic development and per capita cultivated area exert a restraining effect. Developing differentiated emissions reduction strategies to target these key factors is essential for advancing the coordinated development of low-carbon agriculture and food security.

## 1. Introduction

Global warming has emerged as one of the most pressing environmental challenges confronting humanity, and curbing greenhouse gas emissions represents a broad international consensus on addressing climate change. According to the 2024 China Agricultural and Rural Low-Carbon Development Report, total carbon emissions from China’s agricultural sector amount to approximately 793 million tons [1]. Climate warming driven by large-scale greenhouse gas emissions threatens to disrupt food production environments and alter agricultural cropping patterns, ultimately jeopardizing food security. In response, the 2024 “No. 1 Central Document” placed food security at the forefront of national policy, underscoring it as a fundamental cornerstone of both national security and effective governance. Against the backdrop of China’s “dual carbon” targets, achieving a virtuous decoupling between agricultural planting carbon emission intensity (hereinafter “agricultural carbon intensity”) and food security (hereinafter “FSI”)—that is, sustaining continuous improvements in FSI while simultaneously driving down carbon intensity—has become one of the most urgent scientific questions in the field of sustainable agricultural development [2,3,4].

The relationship between agricultural carbon intensity and FSI, however, is far from a simple, unidirectional one. On one hand, a persistent rise in carbon intensity may be rooted in the excessive application of inputs such as chemical fertilizers and pesticides, which progressively degrades cultivated land quality and poses a latent threat to the long-term sustainability of food production. On the other hand, advancing FSI depends, to a considerable degree, on intensifying agricultural inputs, which in turn constrains efforts to reduce carbon intensity. This inherent tension renders the decoupling of agricultural carbon intensity from food security (hereinafter “carbon–food decoupling”) a particularly formidable challenge.

To address these gaps, this study takes the prefecture-level cities of Hunan Province as its research units. By quantifying carbon emissions from agricultural inputs (chemical fertilizers, pesticides, agricultural plastic film, and agricultural diesel), methane emissions from paddy fields, and nitrous oxide emissions from cultivated soil layers, and by employing spatial analysis, LMDI factor decomposition, and the entropy-weight TOPSIS method, this paper systematically investigates the decoupling relationship and driving mechanisms between agricultural carbon intensity and FSI in Hunan Province from 2002 to 2023, with the aim of providing a scientific basis for regional low-carbon agricultural development and emission reductions with efficiency gains.

## 2. Research Status at Home and Abroad

To date, scholars both in China and abroad have conducted extensive empirical research and theoretical discussions on two key themes: carbon sequestration and emissions reduction through agricultural cultivation, and food security. These efforts have yielded substantial findings that provide a solid foundation for future inquiry.

Existing literature on agricultural carbon sequestration and emissions reduction primarily addresses three aspects: the measurement of carbon emissions from agricultural cultivation, the analysis of spatiotemporal patterns, and the identification of influencing factors.

In terms of carbon emission measurement, Qiu et al. [5] applied the emission factor method to estimate carbon emissions from agricultural land use in China between 2004 and 2016; however, inconsistencies in the definition of carbon sources and the selection of emission factors introduced discrepancies in the results. Ma et al. [6] employed a life-cycle approach to construct a carbon emission accounting system for agricultural cultivation; yet, due to the inherent difficulty in precisely defining system boundaries, this method produced uncertainty in the accounting outcomes.

Regarding spatiotemporal evolution, existing studies generally find that agricultural carbon emissions exhibit distinct phased changes and marked regional differentiation. Yang et al. [7], by constructing a carbon balance analysis framework and integrating spatial analysis methods, systematically examined the spatiotemporal evolution of agricultural carbon emissions. Their findings reveal that carbon emissions from grain production in the Gansu section of the Yellow River Basin (2000–2022) followed an “initial increase followed by decrease” trajectory, while carbon uptake has shown a continuous upward trend since 2015. Gao et al. [8] found that carbon uptake in the Huai River Ecological and Economic Belt increased by 16.55% between 2010 and 2022, with the regional carbon budget in a state of dynamic equilibrium, though significant spatial variation in carbon sequestration potential persists across sub-regions. Li et al. [3] and Gong et al. [9] further confirm that agricultural carbon emissions in China exhibit pronounced spatial heterogeneity, with three major net carbon sink growth zones having emerged: Shandong, Henan, and the Northeast.

As for the identification of influencing factors, several scholars have constructed carbon emission factor decomposition models based on the extended Kaya identity, employing methods such as the Logarithmic Mean Divisa Index (LMDI), Monte Carlo simulation, and the STIRPAT model to identify drivers of carbon emissions from agricultural cultivation and quantify each factor’s contribution [10,11,12]. Lin et al. [10], examining carbon emissions in the context of urbanization, conducted an empirical study using the extended Kaya identity and Monte Carlo simulation. They confirmed that economic growth, urbanization, population size, and industrial structure are the core drivers of emission growth, while improvements in energy intensity and adjustments to the energy mix exert a significant inhibitory effect. Zhu et al. [11] and Xu et al. [12], applying the LMDI method, further confirmed that economic output is a positive driver of carbon emissions from energy consumption in China, while reductions in energy intensity exert a significant mitigating effect.

In the field of food security research, scholars both domestically and internationally have primarily focused on three dimensions: the development of food security evaluation indicator systems, the identification of influencing factors, and the evolution of spatial patterns.

With regard to evaluation indicator systems, existing studies predominantly employ either single indicators or multi-dimensional composite indicators to assess food security [13,14,15]. The Food and Agriculture Organization (FAO) of the United Nations has established a comprehensive evaluation framework built on four dimensions: food availability, access, utilization, and stability. Building on this, Chaudhary et al. [13] expanded the framework to seven dimensions and conducted a comprehensive assessment of food security across 156 countries and regions, finding that European and American nations place greater emphasis on food quality, safety, and ecological sustainability. Among domestic scholars, Song et al. [14] constructed a five-dimensional indicator system and applied the entropy-weighted TOPSIS model to evaluate food security in Shandong Province, finding that the Food Security Index (FSI) exhibited a fluctuating upward trend between 2013 and 2022. Gong et al. [15] assessed food security in Henan Province from 2006 to 2013 across three dimensions—production, distribution, and reserves—using the fuzzy matter-element model.

Regarding influencing factors, scholars have employed methods such as the Heckman model and spatiotemporal geographically weighted regression to identify key drivers. It is broadly recognized that natural conditions, agricultural production levels, socioeconomic development, and policy orientation are the core determinants of food security [16,17,18,19,20]. Branković et al. [17] found that agricultural subsidy policies can effectively enhance food production efficiency by incentivizing technological innovation and optimizing management practices, thereby safeguarding food security. Song et al. [18] highlight that the intensity of arable land utilization is a key determinant. Xu et al. [19] and Yang et al. [20] further found that total agricultural machinery power and land reclamation rates exert a bidirectional influence on food security, with this impact exhibiting significant regional variation. Concurrently, scholars have employed index calculation, coupled coordination analysis, and spatial autocorrelation analysis to investigate the spatiotemporal patterns of food security [21,22,23,24]. Overall, while methods for evaluating food security are becoming increasingly diverse, attention to the dynamic evolution of food security under carbon emission constraints remains limited.

Regarding the decoupling relationship between agricultural carbon emissions and food security, directly relevant literature remains scarce; however, a body of research has accumulated on the decoupling between agricultural carbon emissions and economic growth [25,26,27,28,29,30,31,32,33]. Fan et al. [25] and Wang et al. [26], applying the Tapio decoupling model, systematically examined the decoupling relationship between carbon emissions from agricultural land use, economic growth, and food production, revealing a weak decoupling trend between agricultural land use carbon emissions and economic growth. Tian et al. [27] found that agricultural carbon emissions and agricultural economic development in China primarily exhibit two states: weak decoupling and strong decoupling. He et al. [28] combined the Tapio decoupling model with the super-efficiency SBM model to concurrently examine the decoupling relationship and evaluate agricultural performance. Jiang Yan et al. [29] conducted an empirical study in Jiangsu Province, finding that carbon emissions from grain production exhibited strong decoupling from total agricultural output value. While these studies offer valuable methodological insights, a relative shortage of literature directly examining the decoupling relationship between crop cultivation carbon emissions and food security remains.

## 3. Data Sources and Research Methods

### 3.1. General Situation and Regional Division of Research Area

Hunan Province, located in the middle reaches of the Yangtze River, is one of China’s major grain-producing regions and a pilot area for low-carbon agricultural transition. In 2024, the province’s cultivated land area was approximately 4.1488 million hectares, with a total grain output reaching 30.78 million tons. While achieving high grain yields, carbon emissions from rice cultivation accounted for about 40% of the province’s total agricultural carbon emissions. In recent years, the increased reliance on excessive use of pesticides and chemical fertilizers to boost agricultural output has not only led to soil fertility decline and exacerbated agricultural pollution but also significantly contributed to the growth of agricultural carbon emissions. Balancing the need to increase grain production while effectively controlling the rise in agricultural carbon intensity and achieving a benign decoupling between the two has become an urgent scientific challenge.

To facilitate the study, Hunan Province is divided into four regions: The Pinghu areas of northern Hunan, The hilly areas of southern Hunan, High-yielding areas of central and eastern Hunan, and The mountainous areas of western Hunan [30]. The Pinghu areas of northern Hunan includes Changde, Yiyang, and Yueyang, characterized by flat terrain and a high degree of agricultural intensification. The hilly areas of southern Hunan comprises Chenzhou, Yongzhou, and Shaoyang, featuring varied terrain with predominantly terraced and sloped fields. High-yielding areas of central and eastern Hunan consists of Changsha, Xiangtan, Zhuzhou, Loudi, and Hengyang, where basin and hilly landscapes dominate, and grain yield per unit area is relatively high. The mountainous areas of western Hunan includes Huaihua, Zhangjiajie, and the Xiangxi zhou, where farmland fragmentation is particularly notable.

### 3.2. Data Sources

The raw data for estimating agricultural carbon intensity and FSI are primarily sourced from the Hunan Statistical Yearbook and various municipal statistical yearbooks from 2003 to 2024. For individual missing data points, this paper employs linear interpolation to fill in the gaps. Data processing and statistical analysis are conducted using Stata MP 18 (Stata Corp LLC, College Station, TX, USA), and spatial visualization is implemented via ArcGIS 10.8 (Esri, Redlands, CA, USA).

### 3.3. Calculation of Carbon Intensity in Agricultural Production

At present, there is a divergence in the academic community regarding the definition of agricultural carbon sources. According to the existing research results, the carbon sources of agricultural planting are divided into three types: First, methane (CH_4_) emission from rice field irrigation; Second, the destruction of topsoil caused the release of nitrogen oxide (N_2_O); Third, the carbon emissions from the use of agricultural inputs such as fertilizers, pesticides, agricultural films and diesel oil. Carbon emission coefficient method was used to estimate the carbon emission of agricultural planting in Hunan Province. The specific formula is as follows [34]:

(1) $$E_{C}=E_{{CH}_{4}}+E_{N_{2}o}+E_{{CO}_{2}}$$

In the formula, $E_{C}$ represents the total carbon emissions from agricultural planting (10^4^t); $E_{{CH}_{4}}$ represents CH_4_ carbon emissions equivalent from rice cultivation; $E_{N_{2}o}$ represents N_2_O carbon emissions equivalent from tillage (10^4^ t); $E_{{CO}_{2}}$ represents the carbon emissions resulting from the consumption of agricultural inputs (10^4^ t); The calculation formulas are as follows:

(2) $$E_{{CH}_{4}}=\sum S_{i}\times \alpha_{i}\times 27$$

(3) $$E_{N_{2}O}=\sum H_{i}\times \beta_{i}\times 273$$

(4) $$E_{{CO}_{2}}=\sum U_{i}\times \gamma_{i}+D_{ir}\times h+W\times g$$

$S_{i}$ represents the planting area of early rice (i = 1), medium rice (i = 2), and late rice (i = 3) (hm^2^); $H_{i}$ represents the planting area of wheat (i = 1) and corn (i = 2) (hm^2^); $U_{i}$ represents the amounts of usage of agricultural chemical fertilizers, pesticides, agricultural films, and diesel for agricultural use (t); The $\alpha_{i}$ refers to the ${CH}_{4}$ emission factor for rice cultivation; $\beta_{i}$ refers to the emission factor for $N_{2}O$ generated from crops; The $\gamma_{i}$ refers to the carbon emission factor for agricultural inputs; $D_{ir}$ refers to the effectively irrigated area. h refers to the carbon emission factor for farmland irrigation, W refers to the total power of agricultural machinery, and g refers to the carbon emission factor for the total power of agricultural machinery (Table 1); 27 and 273 are the conversion factors for converting $E_{{CH}_{4}}$ and $E_{N_{2}O}$ into CO_2_ equivalents, respectively. The agricultural planting carbon intensity ($E_{CI}$) refers to the ratio of carbon emissions to the sown area of crops.

### 3.4. Measurement of Food Security Based on the Entropy-Weighted TOPSIS Method

Drawing on existing research [38], this paper employs the entropy-weighted TOPSIS method to conduct a comprehensive evaluation of FSI levels. The entropy-weighted method assigns objective weights based on the intrinsic variability of indicator data, thereby effectively reducing the bias introduced by subjective weighting. The TOPSIS method, meanwhile, enables the scientific ranking and comprehensive assessment of multi-indicator decision-making objects. The combination of these two approaches thus enhances the reliability and credibility of the FSI evaluation results. The steps are as follows:

(1) Selection of Food Security Evaluation Indicators

Drawing on previous research [14] and the actual agricultural production conditions of Hunan Province, this study constructs an FSI evaluation indicator system spanning four dimensions: quantitative security, quality security, resource security, and economic security (Table 2). The first dimension, quantitative security, focuses on food production capacity and supply stability. Four indicators are selected: total grain output (C_1_), grain yield per unit of cultivated land (C_2_), per capita grain output (C_3_), and the coefficient of variation in grain output (C_4_). Among these, total grain output reflects a region’s overall supply capacity and serves as the most direct measure of quantitative security; grain yield per unit area and per capita grain output complement this picture from the perspectives of land use efficiency and per capita supply, respectively; and the coefficient of variation captures fluctuations in grain supply over time. Together, these four indicators characterize quantitative security across the dimensions of total volume, efficiency, per capita supply, and stability. The second dimension, quality security, centers on the ecological constraints of agricultural production. Given that excessive fertilizer application degrades both arable land and grain quality, plastic film residues impair soil physical structure, and overuse of pesticides threatens agricultural product safety and the broader ecological environment, three negative indicators are introduced: fertilizer application intensity per unit of arable land (C_5_), plastic film usage intensity (C_6_), and pesticide application intensity (C_7_). Taken together, these indicators reflect the extent to which grain farming adheres to green production standards. The third dimension, resource security, addresses the foundational conditions underpinning grain production. Four indicators are selected: arable land per capita (C_8_), proportion of effectively irrigated area (C_9_), total agricultural machinery power (C_10_), and road density (C_11_). Arable land per capita speaks directly to the resource base available for grain production; the proportion of effectively irrigated area reflects the supportive capacity of agricultural water conservancy infrastructure, which is critical to yield stability; total agricultural machinery power captures the degree of mechanization and its bearing on labor productivity and production efficiency; and road density serves as a proxy for rural infrastructure development, shaping both the supply of agricultural inputs and the efficiency of grain distribution. The fourth dimension, economic security, captures the driving forces and policy environment sustaining grain production. Three indicators are selected: the agricultural output value index (C_12_), the proportion of fiscal expenditure on agriculture (C_13_), and rural per capita disposable income (C_14_). The agricultural output value index reflects the overall development of the agricultural economy and the comprehensive returns of agricultural production; the proportion of fiscal expenditure on agriculture gauges the degree of government support, with public investment serving as an important external safeguard for FSI; and rural per capita disposable income shapes farmers’ incentives to invest in agriculture, bearing directly on the long-term sustainability of grain production.

(2) Data Normalization and Indicator Weight Calculation

To ensure comparability across indicators, all data are normalized prior to weight calculation. The results are presented in Table 2. The normalization formulae are as follows:

(5) $$\mathrm{For}\;\mathrm{positive}\;\mathrm{indicators}:\;x_{ij}^{\prime }=\frac{x_{ij}-{min}_{ij}}{{max}_{ij}-{min}_{ij}}$$

(6) $$\mathrm{For}\;\mathrm{negative}\;\mathrm{indicators}:\;x_{ij}^{\prime }=\frac{{max}_{ij}-x_{ij}}{{max}_{ij}-{min}_{ij}}$$

In the equation, $x_{ij}$ denotes the value of FSI indicator *i* in year j; ${max}_{ij},{min}_{ij}$ represent the maximum and minimum values of indicator i over the study period; $x_{ij}^{\prime }$ denotes the normalized value of $x_{ij}$, where $x_{ij}^{\prime }\in \left[0,1\right]$.

(7) $$P_{ij}=\frac{x_{ij}^{\prime }}{\sum_{j=1}^{n}x_{ij}^{\prime }}\left(j=1,2,\ldots ,n\right)$$

(8) $$e_{j}=-\frac{1}{lnn}\sum_{j=1}^{n}p_{ij}lnp_{ij}$$

where $P_{ij}$ is the comprehensive standardized value; $e_{j}$ is the entropy value of the j-th indicator.

Finally, calculate the indicator weights:

(9) $$W_{j}=\frac{I_{j}}{\sum_{i=1}^{n}I_{j}}\left(j=1,2,\ldots ,n\right)$$

(3) Constructing the weight matrix V

(10) $$V=P\times W={\left[v_{ij}\right]}_{n\times m}$$

Based on the weighting matrix V, the positive ideal solution V^+^ and the negative ideal solution V^−^ are calculated, with the following formulas:

(11) $$\begin{array}{c} V^{+}=\{maxV_{ij}|j=1,2,\ldots ,m\}=\{V_{1}^{+},V_{2}^{+},V_{3}^{+},\ldots ,V_{m}^{+}\}\\ V^{-}=\{minV_{ij}|j=1,2,\ldots ,m\}=\{V_{1}^{-},V_{2}^{-},V_{3}^{-},\ldots ,V_{m}^{-}\} \end{array}$$

(4) Using Euclidean distance, the distances D^+^ and D^−^ between the food security index and the optimal security state V^+^ and the worst-case security state V^−^ are calculated, with the specific formulas as follows:

(12) $$D_{j}^{+}=\sqrt{\sum_{i=1}^{n}{\left(V^{+}-V_{ij}\right)}^{2}}D_{j}^{-}=\sqrt{\sum_{i=1}^{n}{\left(V_{ij}-V^{-}\right)}^{2}}$$

(5) Calculate the proximity of the evaluation object to the ideal solution

(13) $$T_{j}=\frac{D_{j}^{-}}{D_{j}^{+}+D_{j}^{-}}$$

where $T_{j}$ represents the proximity of the FSI level to the optimal security state, with a range of values between 0 and 1; the larger the value, the closer the FSI is to the optimal level.

Furthermore, drawing on the classification method of previous researchers [16], the FSI for each city in Hunan Province is categorized into four levels: when the proximity lies between (0.2, 0.4], FSI is classified as low; between (0.4, 0.5] as lower-middle; between (0.5, 0.6] as higher; and between (0.6, 0.7] as high.

### 3.5. Measuring the Decoupling Effect of Agricultural Planting Carbon Intensity and Food Security

Compared to the OECD decoupling index, the Tapio decoupling index is not affected by the choice of base period. Therefore, this paper adopts the Tapio decoupling index to explore the decoupling relationship between FSI and agricultural carbon intensity [39]. The decoupling model is constructed as follows:

(14) $$\varepsilon =\frac{∆E_{CI}}{E_{CI}^{0}}/\frac{∆T}{T_{0}}$$

In the formula, ε represents the decoupling elasticity index between FSI and carbon intensity, with ${∆E}_{CI}\;and\;E_{CI}^{0}$ denoting the change and base-period value of carbon intensity; $∆T\;and\;T_{0}$ respectively represent the change rate of FSI closeness degree and its base-period value, following Tapio’s decoupling classification framework, this paper categorizes the decoupling relationships between agricultural carbon intensity and FSI for Hunan Province and its cities based on the value range of the decoupling elasticity index ε (Table 3).

### 3.6. Decomposition of Factors Influencing the Decoupling Between Agricultural Carbon Intensity and Food Security

Drawing on existing research [39], this paper applies the Kaya identity in conjunction with the LMDI decomposition model-noted for its high consistency between residual, factor, and total effects—to decompose the elasticity of decoupling between carbon emissions and grain production in agriculture. The decomposition yields five components: energy structure elasticity (ES), energy intensity elasticity (EI), agricultural economic level elasticity (AE), per capita grain sown area elasticity (PG), and rural labor force size elasticity (P).

Each component captures a distinct dimension of agricultural carbon emissions. ES reflects the share of clean energy in total agricultural energy consumption, shaping the level of carbon emissions per unit of energy used. EI measures energy consumption per unit of agricultural output, indicating the efficiency with which energy is used in production. The level of AE reflects the degree of intensification and modernization of the sector, both of which are closely tied to agricultural inputs and associated carbon emissions. PG serves as a proxy for farm operation scale, influencing mechanization levels and carbon emission intensity. Finally, P captures changes in agricultural labor input and is a key factor driving the transformation of production methods. the decomposition model constructed in this paper is as follows:

(15) $$E_{CI}=\frac{E_{CI}}{E}\times \frac{E}{{GDP}_{P}}\times \frac{GDP_{P}}{S}\times \frac{S}{L}\times P$$

Further simplifying Equation (15) yields:

(16) $$E_{CI}=AS\times EI\times AE\times PG\times P$$

In the above two formulas, $E_{CI}$ represents the carbon intensity of agricultural planting, $GDP_{P}$ represents the total output value of agricultural planting, E represents the total agricultural energy consumption, S represents the grain planting area and P represents the scale of rural labor force.

To further explore the contribution of each factor, let carbon intensity in the base period and period t be denoted as $E_{CI}^{0}$ and $E_{CI}^{t}$, respectively. Then, the variation in carbon intensity from the base period to period t is expressed as:

(17) $$∆E_{CI}=E_{CI}^{t}-E_{CI}^{0}=∆AS+∆EI+∆AE+∆PG+∆P$$

In the formula, ∆AS, ∆EI, ∆AE, ∆PG, ∆P represents the contribution of each factor to agricultural planting carbon intensity. The calculation formulas are as follows:

(18) $$∆AS=\sum \frac{E_{CI}^{t}-E_{CI}^{0}}{lnE_{CI}^{t}-lnE_{CI}^{0}}\times \left(lnAS-ln{AS}^{0}\right)$$

(19) $$∆EI=\sum \frac{E_{CI}^{t}-E_{CI}^{0}}{lnE_{CI}^{t}-lnE_{CI}^{0}}\times \left(ln{EI}^{t}-ln{EI}^{0}\right)$$

(20) $$∆AE=\sum \frac{E_{CI}^{t}-E_{CI}^{0}}{lnE_{CI}^{t}-lnE_{CI}^{0}}\times \left(ln{AE}^{t}-ln{AE}^{0}\right)$$

(21) $$∆PG=\sum \frac{E_{CI}^{t}-E_{CI}^{0}}{lnE_{CI}^{t}-lnE_{CI}^{0}}\times \left(ln{PG}^{t}-ln{PG}^{0}\right)$$

(22) $$∆P=\sum \frac{E_{CI}^{t}-E_{CI}^{0}}{lnE_{CI}^{t}-lnE_{CI}^{0}}\times \left(lnP^{t}-lnP^{0}\right)$$

According to Equations (14) and (16), the elasticity index of decoupling between agricultural planting carbon intensity and FSI in Hunan Province is decomposed into energy structure decoupling elasticity ($\varepsilon_{AS}$), energy intensity decoupling elasticity ($\varepsilon_{EI}$), agricultural economy decoupling elasticity ($\varepsilon_{AE}$), per capita grain sown areas decoupling elasticity ($\varepsilon_{PG}$), and rural labor scale decoupling elasticity ($\varepsilon_{P}$), as follows

(23) $$\varepsilon =\;\frac{∆E_{CI}/E_{CI}^{0}}{∆T/T_{0}}=\frac{\left(∆AS+∆EI+∆AE+∆PG+∆P\right)/E_{CI}^{0}}{∆T/T_{0}}\;\;\;\;=\frac{∆\alpha /E_{CI}^{0}}{∆T/T_{0}}+\frac{∆\beta /E_{CI}^{0}}{∆T/T_{0}}+\frac{∆\gamma /E_{CI}^{0}}{∆T/T_{0}}+\frac{∆\delta /E_{CI}^{0}}{∆T/T_{0}}+\frac{∆\eta /E_{CI}^{0}}{∆T/T_{0}}\;=\varepsilon_{AS}+\varepsilon_{EI}+\varepsilon_{AE}+\varepsilon_{PG}+\varepsilon_{P}$$

## 4. Results and Analysis

### 4.1. Spatiotemporal Characteristics of Carbon Intensity in Agricultural Cropping

**Figure 1:**
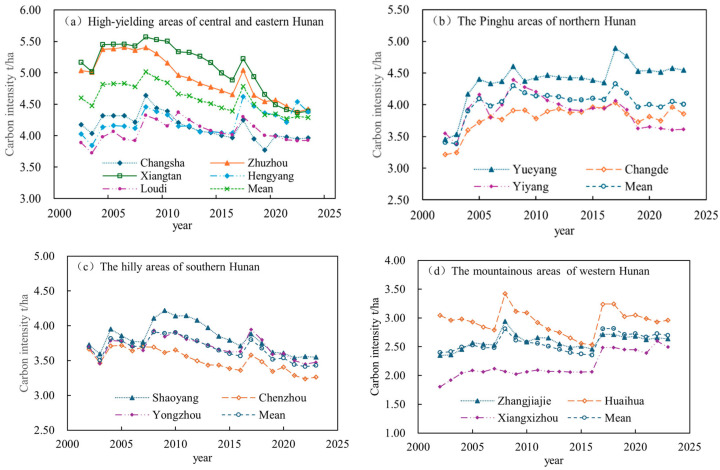
Changes in agricultural carbon intensity by city in Hunan Province from 2002 to 2023.

**Figure 2:**
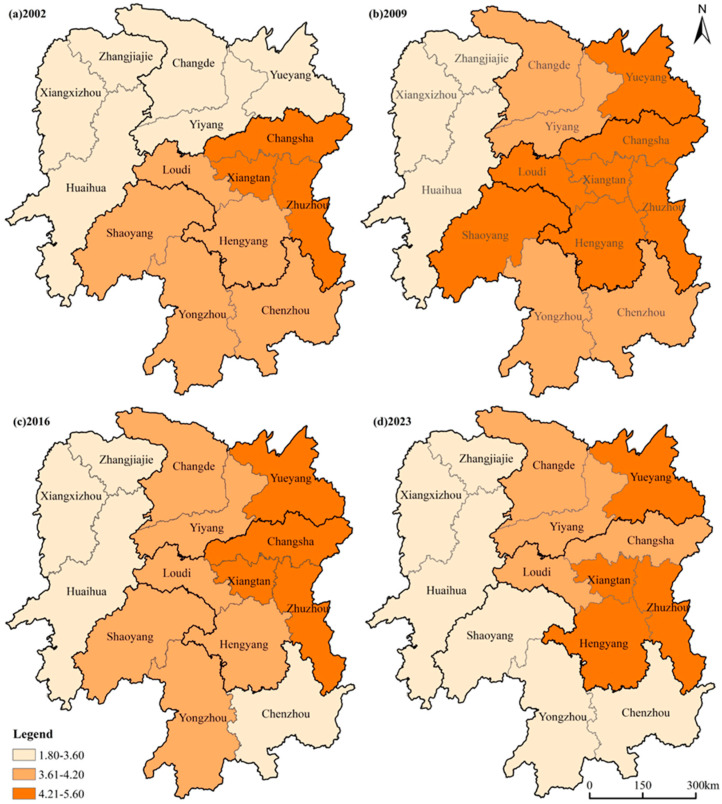
Spatial distribution of agricultural carbon intensity levels across municipalities in Hunan Province.

The spatiotemporal changes in agricultural carbon intensity across Hunan Province’s four regions from 2002 to 2023 are illustrated in Figure 1 and Figure 2. Temporally (Figure 1), agricultural carbon intensity in all regions followed an “M”-shaped trajectory, though the high-yielding areas of central and eastern Hunan and the hilly areas of southern Hunan showed a slight overall downward tendency. Mann–Kendall trend tests (hereinafter “MK tests”) confirmed that this “M”-shaped pattern was statistically significant across all regions. In the high-yielding areas of central and eastern Hunan, mean agricultural carbon intensity peaked in both 2008 and 2017, with cities such as Changsha, Loudi, and Hengyang seeing notable increases between 2002 and 2008 as agricultural intensification advanced—Changsha’s agricultural carbon intensity, for instance, rose from approximately 4.17 t/ha in 2002 to 4.64 t/ha in 2008, before declining steadily by a cumulative 12.8% through 2016 and stabilizing at a relatively low level thereafter, a pattern consistent with the MK test’s finding of a highly significant downward trend. By contrast, the Pinghu areas of northern Hunan, where flat terrain supports a high degree of large-scale farming, maintained persistently elevated agricultural carbon intensity between 3.46 t/ha and 4.52 t/ha with a non-significant upward trend (Z = 0.39, *p* = 0.003); Changde, for example, saw an average annual increase of only 3.60% over the study period. The mountainous areas of western Hunan, constrained by rugged topography that limits large-scale intensive production, recorded the province’s lowest agricultural carbon intensity throughout the study period (2.35–2.81 t/ha), also with a non-significant upward trend (Z = 1.80, *p* = 0.002), though the gradual spread of poverty alleviation initiatives and agricultural mechanization [40] has driven a slow but steady rise. The hilly areas of southern Hunan tell a different story: fragmented farmland combined with a modest recent expansion of cultivated area [41] contributed to a significant decline in agricultural carbon intensity (|Z| > 2.58), with Shaoyang and Chenzhou dropping from 3.88 t/ha and 3.58 t/ha in 2017 to 3.56 t/ha and 3.26 t/ha in 2023, respectively. Overall, agricultural carbon intensity in the hilly areas of southern Hunan falls between that of the mountainous areas of western Hunan and the Pinghu areas of northern Hunan.

From a spatial perspective (Figure 2), agricultural carbon intensity across Hunan Province from 2002 to 2023 exhibited an overall spatial pattern of “higher in the east, lower in the west.” High-value areas (defined using the quantile method, with the top 30% of samples classified as high-value zones, the bottom 30% as low-value zones, and the remainder as mid-value zones) were concentrated primarily in the high-yielding areas of central and eastern Hunan and the Pinghu areas of northern Hunan, while low-value areas were largely confined to the mountainous areas of western Hunan. Moran’s I analysis (Table 4) showed that values across all years during the study period ranged from 0.393 to 0.578 (*p* < 0.01), indicating significant positive spatial autocorrelation in agricultural carbon intensity across the province, with both high- and low-value areas forming contiguous clusters. In the high-yielding areas of central and eastern Hunan, 60% of prefectural-level cities recorded high agricultural carbon intensity values in 2002 (Figure 2a), likely linked to the heavy inputs of fertilizers and pesticides and frequent mechanized operations associated with the pursuit of high yields during that period [30]. By 2009, carbon intensity in this region had risen overall compared to 2002, with Loudi and Hengyang shifting from mid-value to high-value zones. By 2016, the Changsha-Zhuzhou-Xiangtan metropolitan area had reached high-value levels across the board, though by 2023 Changsha had retreated to the mid-value range while Xiangtan, Zhuzhou, and Hengyang remained in the high-value zone. In the Pinghu areas of northern Hunan, all cities fell within the low-value zone in 2002; from 2009 to 2023 the spatial pattern remained broadly stable, with most cities at mid-value levels except Yueyang, which stayed in the high-value zone. In the hilly areas of southern Hunan, agricultural carbon intensity in Shaoyang, Yongzhou, and Chenzhou declined continuously throughout 2002–2023, with all three cities moving from the mid-value zone in 2002 to the low-value zone by 2023. Shaoyang alternated between mid- and high-value levels from 2002 to 2016, possibly reflecting fluctuations in agricultural input use and uneven implementation of green transition policies during that period [42], while Chenzhou and Yongzhou entered the low-value zone in 2016 and 2023, respectively, and have remained stable since. The mountainous areas of western Hunan, constrained by terrain that has kept farmland fragmented and large-scale farming limited, consistently maintained low levels of agricultural input intensity and thus sustained low agricultural carbon intensity values throughout the study period.

### 4.2. Spatiotemporal Evolution Characteristics of Food Security

**Figure 3:**
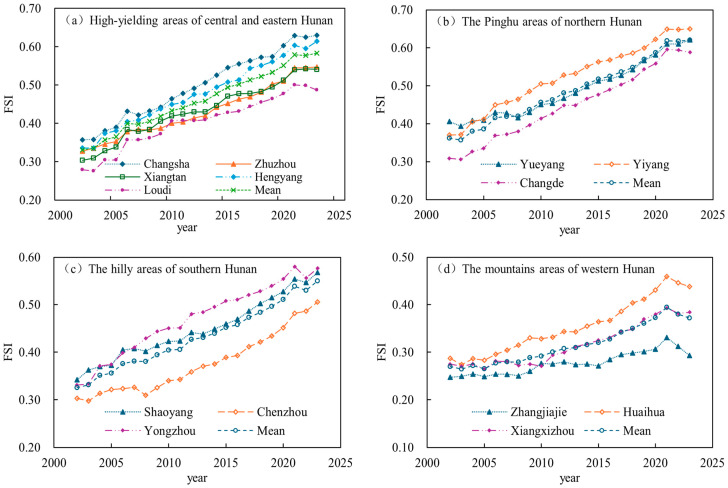
Time-series changes in the FSI across cities of Hunan Province from 2002 to 2023.

**Figure 4:**
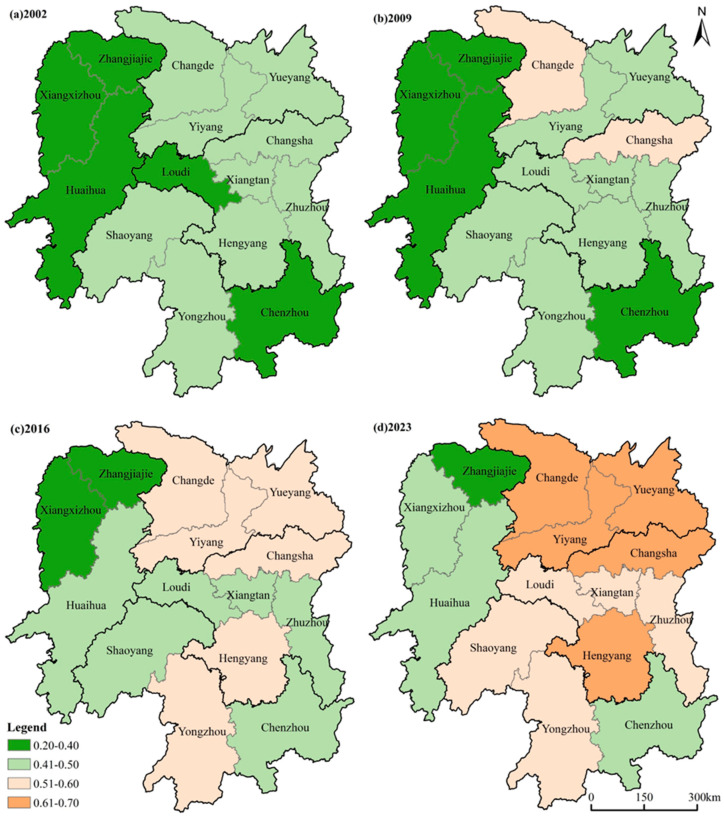
Spatial distribution of FSI grades across cities in Hunan Province from 2002 to 2023.

The spatiotemporal changes in the FSI across Hunan Province’s four regions from 2002 to 2023 are shown in Figure 3 and Figure 4. Temporally (Figure 3), the FSI in all regions followed a sustained upward trend over the study period. The high-yielding areas of central and eastern Hunan, the Pinghu areas of northern Hunan, and the mountainous areas of western Hunan all peaked in 2021 before declining slightly, while the hilly areas of southern Hunan maintained a fluctuating yet consistently upward trajectory throughout. MK tests confirmed that the upward trends across all regions were statistically significant (|Z| > 2.58, *p* < 0.01). In the high-yielding areas of central and eastern Hunan, the mean FSI rose from 0.33 in 2002 to 0.56 in 2023 (Z = 6.37, *p* < 0.01), with most cities following a pattern similar to that of Changsha, where the FSI dipped slightly after 2021. In the Pinghu areas of northern Hunan, flat terrain and favorable water and soil conditions supported the most pronounced regional improvement, with the mean FSI climbing from 0.36 in 2002 to 0.62 in 2023 (Z = 6.37, *p* < 0.01); Yiyang recorded the sharpest increase, rising from 0.37 to 0.65—a gain of 75.7%. In the hilly areas of southern Hunan, abundant light and heat resources, combined with the upgrading of low- and medium-yield farmland [40], drove a substantial overall rise in FSI, with Yongzhou’s index growing from 0.33 in 2002 to 0.57 in 2023, a 73% increase. The mountainous areas of western Hunan, long constrained by rugged terrain, maintained the province’s lowest FSI levels (0.25–0.46) for much of the period; however, poverty alleviation programs, rural revitalization efforts, and the adoption of modern agricultural technologies [42] have accelerated growth in recent years (Z = 6.03, *p* < 0.01). Huaihua’s FSI, for instance, jumped from 0.29 in 2015 to 0.45 in 2023, substantially offsetting the limiting effect of the natural environment on FSI.

From a spatial perspective (Figure 4), the FSI across Hunan Province’s cities from 2002 to 2023 showed an overall pattern of broad improvement alongside a persistent north–south gradient, with high-value areas concentrated in the Pinghu areas of northern Hunan and low-value areas remaining largely confined to the mountainous areas of western Hunan and the hilly areas of southern Hunan. Moran’s I analysis (Table 5) revealed consistently positive spatial autocorrelation throughout the study period, with values rising from 0.089 in 2002 to 0.376 in 2023, all significant at Pless than 0.05. This indicates that the spatial clustering of FSI values, both high and low, strengthened progressively over time. In the high-yielding areas of central and eastern Hunan, all cities recorded medium-to-low FSI levels in 2002, with Changsha the first to reach a higher level by 2009. Growth slowed somewhat in 2016 due to adjustments in arable land structure [43], but by 2023 the entire region had reached high or higher levels. In the Pinghu areas of northern Hunan, all cities similarly started at medium-to-low levels in 2002; Changde entered the higher-level category by 2009 and Yiyang by 2016, and their radiating effect on surrounding areas helped bring the entire region to high levels by 2023. In the hilly areas of southern Hunan, FSI levels were uniformly low in 2002, yet by 2023 the entire region had entered the higher-level category, driven in large part by the promotion of double-cropping rice and dryland farming in cities such as Yongzhou and Shaoyang [43,44]. The mountainous areas of western Hunan, by contrast, have long remained at medium-to-low levels; fragmented farmland and limited agricultural scale have constrained FSI growth, making this region’s improvement the most modest of the four.

### 4.3. Evolution of the Decoupling Status Between Agricultural Carbon Intensity and Food Security

**Figure 5:**
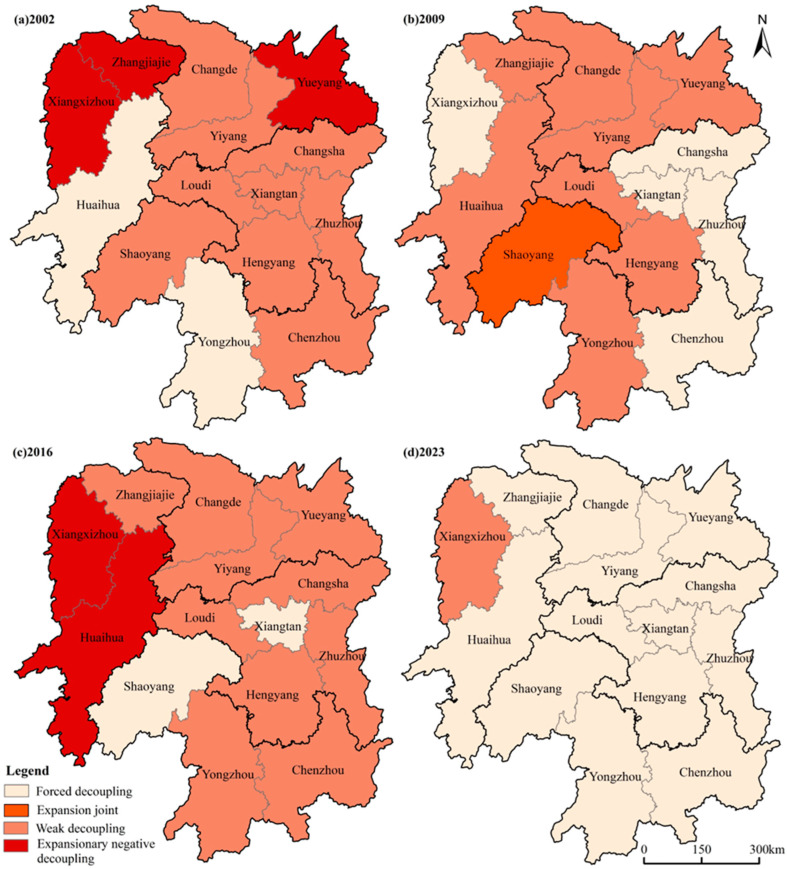
Spatial distribution of decoupling status across municipalities in Hunan Province.

Table 6 and Figure 5 illustrate the evolution of the decoupling relationship between agricultural carbon emissions and FSI across Hunan Province’s municipalities from 2002 to 2023. Over the study period, this relationship shifted from being predominantly weak to largely strong across the entire province. Between 2002 and 2009, the number of municipalities exhibiting strong decoupling grew from 2 to 5, with the corresponding proportion rising from 14.3% to 35.7%. Between 2009 and 2016, however, the decoupling relationship became less stable: the number of strongly decoupled municipalities dropped to two, while those showing weak decoupling and expanding negative decoupling each increased by two, suggesting that some municipalities, Changsha among them, were achieving higher grain yields at the cost of significantly higher carbon emissions. Between 2016 and 2023, the number of strongly decoupled municipalities rose sharply to 13, a 5.5-fold increase from 2016, while municipalities showing expanding linkage or expanding negative decoupling fell to zero by 2023, indicating that a broadly positive decoupling of carbon emissions from FSI has been achieved across the province.

From a spatial perspective, the decoupling of agricultural carbon emissions and FSI in Hunan Province exhibits spatiotemporal characteristics of “overall improvement with significant regional divergence” (Figure 5). Moran’s I results (Table 7) show that the spatial clustering of carbon–food decoupling across the province fluctuated markedly between 2002 and 2023, with most years passing significance tests, indicating that the decoupling status of prefectural-level cities is not randomly distributed but instead displays phased clustering and divergence. In the high-yielding areas of central and eastern Hunan, all cities were in a state of weak decoupling in 2002, as green agricultural development policies and low-carbon technologies had yet to be widely adopted. By 2009, following the State Council’s No. 1 Central Document strengthening arable land protection policies and the promotion of advanced agricultural technologies, FSI in the region rose steadily, driving the Changsha-Zhuzhou-Xiangtan area from weak to strong decoupling. In 2016, decoupling in this region regressed: with the exception of Xiangtan, which maintained strong decoupling, the remaining cities increased agricultural inputs to pursue higher FSI, causing agricultural carbon intensity to rise and decoupling to weaken. By 2023, however, the entire region had returned to strong decoupling. In the hilly areas of southern Hunan, fragmented farmland in Yongzhou and Chenzhou hindered large-scale production between 2002 and 2016, leaving both FSI and agricultural carbon intensity relatively unstable and causing the decoupling state to alternate between strong and weak. By 2023, changes in crop structure [40] had significantly improved agricultural production efficiency across the region, enabling it to achieve strong decoupling throughout. In the Pinghu areas of northern Hunan, between 2002 and 2009, Yueyang shifted from expanding negative decoupling to weak decoupling, while Changde and Yiyang remained in weak decoupling. From 2009 to 2023, the entire region transitioned from weak to strong decoupling. In the mountainous areas of western Hunan, Huaihua leveraged the flat terrain of the Yuanma Basin between 2002 and 2009 to achieve economies of scale, supporting strong decoupling. Xiangxi zhou and Zhangjiajie, by contrast, faced severely fragmented terrain and extensive farming practices that caused agricultural carbon intensity to grow faster than FSI gains, resulting in expanding negative decoupling during this phase. Between 2009 and 2016, both Xiangxi Prefecture and Huaihua experienced a rebound in decoupling, reflecting a fluctuating balance between FSI-driven agricultural expansion and the transition to low-carbon practices. By 2023, all cities in the region had achieved strong decoupling except Xiangxi zhou, which remained in weak decoupling due to its specialized agriculture’s dependence on high fertilizer inputs and persistent topographical constraints.

### 4.4. Factor Analysis of the Decoupling Between Agricultural Carbon Intensity and Food Security

To identify the contributing factors behind the decoupling of carbon emissions and FSI in agricultural cultivation, this study applied the LMDI method to decompose the carbon–food decoupling elasticity into five components: ES elasticity, EI elasticity, AE elasticity, PG elasticity, and P elasticity. The results are presented in Table 8.

The cumulative contributions of EI, ES, and P were −196.42, −24.54, and −9.27, accounting for 85.3%, 10.7%, and 4.0% of the total promoting effects, respectively, all of which passed Bootstrap significance tests (*p* < 0.05). Among these, EI had the most pronounced promoting effect (*p* < 0.001): as agricultural mechanization advanced and energy-saving technologies spread, energy consumption per unit of agricultural output declined steadily, lowering carbon emission intensity and causing total carbon emissions to grow more slowly than FSI, thereby driving decoupling. The optimization of ES (*p* = 0.0092) gradually raised the share of clean energy in agricultural production, reducing carbon emissions per unit of energy consumed and further reinforcing the decoupling effect. The sustained contraction of P (*p* = 0.0421) reduced some labor-intensive agricultural activities, lowering overall energy demand and contributing modestly to decoupling.

On the inhibiting side, the cumulative decoupling effects of AE and PG were 215.81 and 28.85, accounting for 88.2% and 11.8% of the total inhibiting effects, respectively, both passing Bootstrap significance tests (*p* < 0.001 and *p* = 0.0206). AE had the strongest inhibiting effect: as the agricultural economy grew while FSI gains remained relatively limited, demand for agricultural products became increasingly rigid and diversified, pushing up food production costs, agricultural material consumption, and agricultural carbon intensity [45], thereby slowing the decoupling process in Hunan Province. The expansion of PG similarly raised both FSI and agricultural input use, exerting an additional inhibiting effect on decoupling.

At the sub-regional level, the drivers of carbon–FSI decoupling varied considerably across regions (Table 8). In the high-yielding areas of central and eastern Hunan, the overall effect was −2.22, indicating that carbon emissions grew more slowly than FSI, reflecting a relatively favorable decoupling trajectory. EI was the dominant promoting factor, contributing −56.34 and accounting for 83.60% of the region’s total promoting effect, as sustained improvements in energy use efficiency reduced carbon emission intensity per unit of output while FSI remained stable. P and ES contributed −5.71 and −5.34, accounting for 8.47% and 7.92%, respectively, indicating that labor transfer to non-farm sectors and a rising share of clean energy also played meaningful roles. AE, with a contribution of 61.80, accounting for 96.96% of the total inhibiting effect, was the primary factor restraining decoupling in this region.

In the Pinghu areas of northern Hunan, the overall effect was 6.54, meaning carbon emissions grew faster than FSI, making this the sub-region under the greatest decoupling pressure among the four. Although EI remained the main promoting factor with a contribution of −32.11, the combined inhibiting effects of AE, P, and PG totaled 38.66, preventing meaningful improvement in the decoupling state. Notably, P acted as an inhibiting factor here, contributing 3.98 and accounting for 10.29% of the total inhibiting effect, reflecting the fact that traditional farming practices in the Dongting Lake Plain have yet to undergo fundamental transformation, a key reason why decoupling in this region has lagged behind others.

In the hilly areas of southern Hunan, the overall effect was −2.05, indicating that carbon emissions grew more slowly than FSI, making this the sub-region with the most well-established decoupling mechanism among the four. EI, P, and ES accounted for 82.49%, 10.20%, and 7.32% of the promoting effects, respectively, showing that the favorable decoupling trajectory here was driven primarily by declining EI, with P contraction and ES optimization also contributing. Although AE exerted some resistance, contributing 42.71 and accounting for 93.81% of the total inhibiting effect, the promoting forces ultimately prevailed, enabling the region to achieve overall carbon–food decoupling.

In the mountainous areas of western Hunan, the overall effect was 12.92, indicating that carbon emissions grew faster than FSI, making this the sub-region facing the most severe decoupling challenge among the four. AE and PG were the primary inhibiting factors, contributing 81.09 and 21.14 and accounting for 79.32% and 20.68% of the total inhibiting effect, respectively. The relatively high share of PG compared to other sub-regions may reflect the continued development of sloped farmland and the rapid expansion of grain cultivation in recent years. EI and ES contributed −69.32 and −17.29, respectively, and their promoting effects were stronger here than in the other three sub-regions; however, these gains were insufficient to offset the combined pressure of AE expansion and PG growth, and the decoupling situation in the mountainous areas of western Hunan warrants continued close attention.

**Figure 6:**
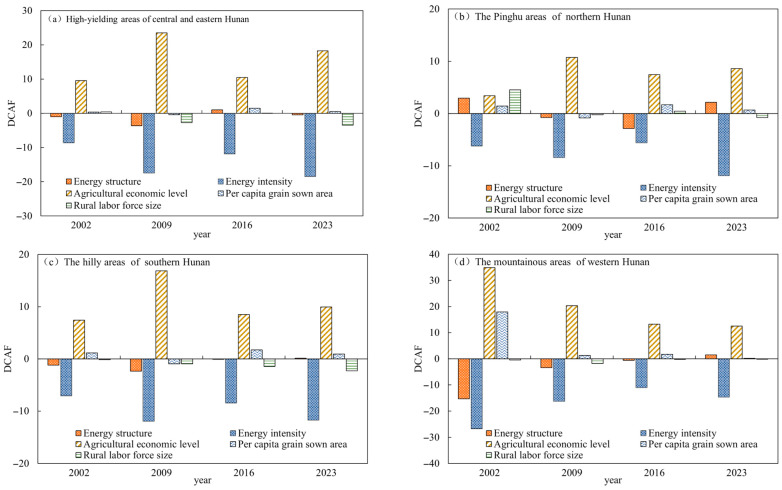
Interannual variations in contributions of factors influencing carbon–food decoupling across regions in Hunan. Note: DCAF denotes the decoupling elasticity contribution value.

Looking at the interannual variations in influencing factors (Figure 6), the impact of AE and EI on the decoupling of agricultural carbon intensity and FSI remains relatively stable. Notably, the elasticity of AE performance was positive in every year across Hunan Province, indicating that it is the primary factor suppressing carbon–food decoupling in the province’s various districts, implying that economic growth is accompanied by an increase in carbon emissions. The decomposition elasticity of EI was negative in all years, making it the core factor in carbon–food decoupling. This may be due to the adjustment of Hunan Province’s agricultural crop structure and the adoption of more environmentally friendly agricultural technologies [41], which led to a significant reduction in EI, thereby promoting carbon–food decoupling. The direction of influence of ES, P and PG, however, alternated across different years. From 2002 to 2023, the effect of P on carbon–food decoupling in the high-yielding areas of central and eastern Hunan alternated between positive and negative, while the ES showed negative decoupling elasticity values in all years except for a positive value in 2016. In the Pinghu areas of northern Hunan, the inhibitory effect of PG on the carbon–food decoupling showed a weakening trend, though its elasticity value remained positive by 2023; meanwhile, the elasticity of ES exhibited a U-shaped trend. Although the decoupling elasticity of ES was positive in 2023, its overall effect remained negative (Table 5), indicating that ES generally promoted carbon–food decoupling in northern Hunan’s Pinghu areas. In the hilly areas of southern Hunan, the decomposed elasticity values of ES also showed a U-shaped trend, but with smaller fluctuations compared to those in northern Hunan’s Pinghu areas. The decomposed elasticity of P exhibited considerable positive–negative fluctuations; however, its inhibitory effect on carbon–food decoupling weakened over time. In the mountainous areas of western Hunan, the decomposed elasticity of ES turned from negative to positive in 2016, indicating that ES’s role shifted from promoting to inhibiting carbon–food decoupling. This shift may be attributed to a resurgence in the share of high-carbon energy sources, lagging clean energy substitution, and the high-carbon characteristics of agricultural energy consumption [46,47].

## 5. Discussion

### 5.1. Spatiotemporal Changes in Agricultural Carbon Intensity and Food Security in Hunan Province

Existing research has confirmed that spatiotemporal variation in agricultural carbon intensity in China is primarily driven by natural conditions and the level of agricultural intensification: plain areas tend to become high-value carbon emission zones due to large-scale farming and heavy agricultural input use, while mountainous and hilly areas tend to form low-value zones owing to smaller cultivation scales and lower input intensity [3]. This study finds that agricultural carbon intensity in Hunan Province follows a spatial pattern of “higher in the east, lower in the west,” with the high-yielding areas of central and eastern Hunan and the Pinghu areas of northern Hunan as high-value zones, and the hilly areas of southern Hunan and the mountainous areas of western Hunan as low-value zones, broadly consistent with existing findings [44]. One distinction is that prior studies have largely examined the evolution of agricultural carbon intensity at the provincial scale, whereas this study divides Hunan Province into four sub-regions and reveals spatiotemporal dynamics and regional differences at the prefectural level, addressing a gap in research at this scale.

Existing research has also shown that FSI is closely associated with regional agricultural production foundations and water–soil resource conditions. Plain areas typically exhibit higher FSI due to large-scale farming, high-standard farmland construction, and well-developed irrigation infrastructure, while mountainous and hilly areas tend to have relatively lower FSI owing to topographic constraints [19,20]. This study finds that FSI in Hunan Province shows a pattern of “broad-based improvement with a south-to-north gradient,” with the Pinghu areas of northern Hunan maintaining higher FSI levels and the mountainous areas of western Hunan remaining at medium-to-low levels over the long term, consistent with the findings of Song et al. [14] and Gong et al. [15]. It should be noted that this study focuses primarily on the spatiotemporal changes in FSI across sub-regions and their influencing factors, with limited attention to differentiated pathways for improving FSI, which will be a key direction for future research.

### 5.2. Spatiotemporal Changes and Driving Mechanisms of Carbon–FSI Decoupling in Hunan Province

Existing studies on the decoupling of agricultural carbon emissions and FSI have focused primarily on the relationship between agricultural carbon emissions and economic growth, with multiple Tapio model-based studies finding a weak decoupling relationship between the two [25,26,27]. This study finds that carbon–FSI decoupling in Hunan Province evolved from “predominantly weak decoupling” through a “surge in strong decoupling” to province-wide strong decoupling, consistent with existing findings on the phased evolution of decoupling [25,29]. The four sub-regions, however, differed markedly in their decoupling trajectories and in the direction and magnitude of driving factor contributions, suggesting that relying solely on provincial averages masks localized carbon emission pressures and decoupling potential. Furthermore, by extending the decoupling analysis from the provincial scale to the prefectural and agricultural sub-regional scales, this study finds that carbon–FSI decoupling in Hunan Province is characterized by “overall improvement, a winding process, and regional asynchrony,” a finding similar to that of Fan et al. [25], providing a more granular scientific basis for regionally differentiated policy interventions.

On the driving factors side, existing studies have largely focused on identifying decoupling states [27,29], with relatively limited quantitative analysis of the underlying mechanisms. This study applies the LMDI decomposition method to break down carbon–FSI decoupling elasticity into five factors, ES, EI, AE, PG, and P, and finds that EI is the core factor promoting decoupling, AE is the primary inhibiting factor, and the inhibiting effect of PG is particularly pronounced in the mountainous areas of western Hunan. Notably, some studies [5,12] have argued that labor transfer weakens food production capacity and intensifies carbon–food tensions, whereas this study finds that the contraction of P has generally promoted decoupling, a divergence that may be related to differences in agricultural mechanization levels and industrial structures across study regions, and warrants further investigation. These findings deepen the understanding of the driving mechanisms behind agricultural carbon–FSI decoupling and provide a reference for formulating regionally differentiated carbon–food coordination strategies.

While this study offers improvements in analytical scale and driving mechanism identification, certain limitations remain. First, the emission factor method relies on fixed coefficients and cannot capture the dynamic variation in emission factors across different regions and production conditions, which may introduce some estimation bias. Second, while the LMDI decomposition method can identify the direction and magnitude of each factor’s contribution, it cannot capture interaction effects or nonlinear relationships among factors. In addition, data availability constraints required the use of proxy variables for some indicators, which may affect the precision of the results. Future research could be extended in the following directions. First, in terms of methodology, spatial econometric models or panel threshold models could be introduced to explore the nonlinear effects and spatial spillover effects of driving factors, complementing the limitations of the LMDI approach. Second, in terms of research scope, sub-sectors such as livestock farming and aquaculture could be incorporated into the analytical framework to build a more comprehensive carbon–food co-accounting system for agriculture. Third, in terms of policy alignment, combining policy scenarios such as high-standard farmland construction and agricultural green development pilot programs, future studies could evaluate the effectiveness of differentiated regulatory pathways in achieving carbon–food coordination goals.

## 6. Conclusions

This study takes the cities of Hunan Province as the research unit. By measuring carbon intensity and the FSI, and employing the Tapio decoupling model and the LMDI decomposition method, it systematically investigates the spatiotemporal changes and driving factors of the decoupling of agricultural carbon intensity and FSI in agriculture from 2002 to 2023. The main conclusions are as follows: (1) The agricultural carbon intensity across the province generally presents a pattern of being “high in the east and low in the west.” High-value areas of agricultural carbon intensity are mainly concentrated in the high-yielding areas of central and eastern Hunan and the Pinghu areas of northern Hunan, while low-value areas are focused in the mountainous areas of western Hunan. (2) The FSI shows a trend of being “high in the northeast and low in the southwest.” The FSI is relatively high in the Pinghu areas of northern Hunan and the high-yielding areas of central and eastern Hunan, at a medium-low level in the mountainous areas of western Hunan, and falls between these levels in the hilly areas of southern Hunan. (3) The decoupling of carbon–food decoupling in agriculture has undergone a phased evolution from “weak decoupling” to “strong decoupling.” By 2023, cities exhibiting strong decoupling accounted for 92.9% of the province, although the evolution paths of decoupling varied across different regions. (4) Significant spatial heterogeneity exists in the effects of factors influencing carbon–food decoupling. EI is the most core positive driving factor affecting carbon–food decoupling, with its impact being most prominent in the mountainous areas of western Hunan. Conversely, the AE is the main inhibiting factor for carbon–food decoupling, with its effect being evident in the high-yielding areas of central and eastern Hunan and the mountainous areas of western Hunan. Except for their inhibitory effects in Changde and Yueyang within the Pinghu areas of northern Hunan, ES and P act as promoting factors in other regions. The PG generally exhibits an inhibitory effect, which is particularly significant in the mountainous areas of western Hunan. (5) Based on the above conclusions, the following differentiated policy recommendations are proposed. In the Pinghu areas of northern Hunan, where large-scale farming drives significant carbon emission pressure, priority should be given to promoting water-saving irrigation and precision fertilization technologies to reduce excessive agricultural input use. In the high-yielding areas of central and eastern Hunan, where food production intensity is high, efforts should focus on accelerating the upgrading of energy-efficient agricultural machinery to lower energy consumption per unit of output. In the hilly areas of southern Hunan, which have achieved the best decoupling performance, the focus should be on consolidating existing energy-saving and emission-reduction gains to prevent carbon emission rebound driven by agricultural economic expansion. In the mountainous areas of western Hunan, where farmland conditions are poor and crop yields per unit area remain low, priority should be given to advancing high-standard farmland construction to improve land productivity, while also expanding the use of clean energy in agricultural production.

## Figures and Tables

**Table 1:** Carbon emission factors for agricultural inputs.

Agricultural Inputs	Carbon Emission Factor	Data Source	Agricultural Inputs	Carbon Emission Factor	Data Source
Chemical fertilizer	895.60 (kg/t)	[35]	Agricultural diesel	592.70 (kg/t)	[36]
Pesticides	4934.10 (kg/t)		Effective irrigated area	266.48 (kg/hm^2^)	[35,37]
Total agricultural machinery power	0.18 (kg/kW)		Agricultural film	5180.00 (kg/t)	[37]

**Table 2:** Hunan Province food security evaluation indicators and their weights.

Primary Indicator	Weight of Primary Indicators/%	Secondary Indicators	Unit	Nature	Weight of Second Indicators/%
Quantity Security	25.06	Grain production ($C_{1}$)	10^4^ t	+	10.88
		Grain yield per unit of cultivated land area ($C_{2}$)	kg/ha	+	6.34
		Per capita grain yield ($C_{3}$)	kg/per	+	5.28
		Coefficient of variation in grain yield (standard deviation of grain yield over five years/average grain yield over five years) ($C_{4}$)	%	−	2.57
Quality Security	13.95	Fertilizer application per unit of cultivated land area ($C_{5}$)	t/ha	−	4.66
		Agricultural film usage per unit of cultivated land area ($C_{6}$)	t/ha	−	4.01
		Pesticide application per unit of cultivated land area ($C_{7}$)	t/ha	−	5.29
Resource Security	32.50	Per capita arable land area ($C_{8}$)	ha/per	+	4.87
		Proportion of effectively irrigated area ($C_{9}$)	%	+	7.35
		Total agricultural machinery power ($C_{10}$)	10^4^ kw	+	10.19
		Road network density ($C_{11}$)	km/ha	+	10.10
Economic Security	28.49	Agricultural output value index ($C_{12}$)	%	+	4.18
		Percentage of Agricultural Expenditure ($C_{13}$)	%	+	8.23
		Rural per capita disposable income ($C_{14}$)	10^4^ yuan	+	16.05

**Table 3:** Tapio decoupling elasticity coefficient status and its implications.

Status	Disengaged State	$∆E_{CI}/E_{CI}^{0}$	$∆T/T^{0}$	Decoupling Resilience	Meaning
Negative Decoupling	Negative decoupling expansion	>0	>0	e>1.2	$E_{CI}$ increase, T increases, but the rate of increase $E_{CI}$ is less than that for T.
	Negative decoupling expansion	>0	<0	e<0	$E_{CI}$ decreases, T increases
	Weak negative decoupling	<0	<0	0≤e<0.8	$E_{CI}$ decreases, T decreases, but $E_{CI}$ decreases at a faster rate than T.
Connect	Expansion joint	>0	>0	0.8≤e≤1.2	$E_{CI}$ increased, T increased, but the growth rates of both were broadly consistent.
	Deteriorating connections	<0	<0	0.8≤e≤1.2	$E_{CI}$ decreases, T decreases, but the rate of decrease for both is essentially the same.
Decoupling	Degrowth decoupling	<0	<0	e>1.2	$E_{CI}$ decreases, T decreases, but $E_{CI}$ decreases at a lower rate than T.
	Weak decoupling	>0	>0	0≤e<0.8	$E_{CI}$ increases, T increases, but the rate of increase $E_{CI}$ exceeds that of T.
	Forced decoupling	<0	>0	e<0	$E_{CI}$ increased, T decreased

Note: $E_{CI}$ denotes the agricultural carbon intensity of agricultural cultivation, and *T* represents the level of FSI.

**Table 4:** Global Moran’s Index (Moran’s I) and test of agricultural carbon emission intensity in Hunan Province from 2002 to 2023.

	2002	2006	2009	2012	2016	2019	2023
Moran’s I	0.578 **	0.535 **	0.525 **	0.507 **	0.512 **	0.450 **	0.446 **

Note: ***, **, and * denote significance at the 1%, 5%, and 10% levels, respectively.

**Table 5:** Global Moran’s Index (Moran’s I) and Significance Tests for the FSI in Hunan Province from 2002 to 2023.

	2002	2006	2009	2012	2016	2019	2023
Moran’s I	0.089 **	0.211 *	0.169 *	0.200 *	0.233 *	0.240 *	0.376 **

Note: ***, **, and * denote significance at the 1%, 5%, and 10% levels, respectively.

**Table 6:** Number and proportion of each decoupling status across prefecture level cities in Hunan.

Disengaged State	2002		2009		2016		2023	
	Number of Cities (n)	Ratio (%)	Number of Cities (n)	Ratio (%)	Number of Cities (n)	Ratio (%)	Number of Cities (n)	Ratio (%)
FD	2	14.3	5	35.7	2	14.3	13	92.9
WD	9	64.3	8	57.2	10	71.4	1	7.1
EJ	0	0.0	1	7.1	0	0.0	0	0.0
NDE	3	21.4	0	0.0	2	14.3	0	0.0

Note: FD denotes forced decoupling, WD denotes weak decoupling, EJ denotes expansion joint, and NDE denotes negative expansion decoupling.

**Table 7:** Global Moran’s Index (Moran’s I) and Significance Tests for the Carbon–Food Decoupling Index in Hunan Province from 2002 to 2023.

	2002	2006	2009	2012	2016	2019	2023
Moran’s I	−0.038 *	−0.227 *	0.292 *	0.190 *	−0.170 *	0.273 *	0.376 **

Note: ***, **, and * denote significance at the 1%, 5%, and 10% levels, respectively.

**Table 8:** Driving effects of factors contributing to the decoupling of agricultural carbon intensity from food security in Hunan Province.

Zoning	City	Energy Structure	Energy Intensity	Agricultural Economic Level	Per Capita Grain Sown Area	Rural Labor Force Size	Overall Effect
High-yielding areas of central and easter Hunan	Changsha	−2.67	−11.05	13.39	0.78	−0.96	−0.51
	Zhuzhou	−0.97	−12.84	14.55	−0.53	−1.53	−1.31
	Xiangtan	−0.96	−11.50	12.65	0.06	−1.57	−1.2
	Hengyang	−0.09	−8.62	9.36	0.68	−0.58	0.75
	Loudi	−0.65	−12.33	11.85	0.95	−1.07	0.05
The Pinghu areas of norther Hunan	Changde	0.45	−10.78	9.13	1.73	0.62	1.14
	Yiyang	−0.24	−9.18	8.80	1.04	−0.31	0.11
	Yueyang	1.28	−12.15	12.30	0.19	3.67	5.29
The hilly areas of southern Hunan	Chenzhou	−2.09	−12.87	15.39	0.29	−1.50	−0.79
	Yongzhou	−0.83	−12.70	12.87	1.24	−1.72	−1.14
	Shaoyang	−0.56	−13.66	14.45	1.29	−1.63	−0.12
The mountainous areas of western Hunan	Zhangjiajie	−4.92	−28.40	32.89	6.33	−2.37	3.52
	Huaihua	−3.39	−13.58	14.48	2.00	−0.21	−0.70
	Xiangxizhou	−8.98	−27.34	33.72	12.81	−0.11	10.10
Total		−24.54	−196.42	215.81	28.85	−9.27	15.06

## Data Availability

The original contributions presented in this study are included in the article. Further inquiries can be directed to the corresponding author.

## References

[1] Chen Y., Dai X.W. (2025). A Study on carbon emissions from planting industry and its decoupling with grain production in major grain-producing regions of China. Res. Environ. Sci..

[2] Mei Y., Dai L.Z., Wu M.Y. (2024). Research on the coupling and coordination of agricultural carbon emission efficiency and food security in Anhui Province. J. Saf. Environ..

[3] Li G.Z., Kang D.L. (2025). Spatiotemporal distribution, decoupling characteristics, and driving fac-tors of agricultural carbon emissions in Hebei. North. Hortic..

[4] Dou C.X., Liu X.Z., Liu J.X. (2025). Review on the coordinated development of carbon sequestration and emissions reduction in agricultural planting and food security. Chin. J. Eco-Agric..

[5] Qiu Y.H., Wang P., Su S.P. (2019). Temporal and spatial evolution characteristics and driving factors of carbon emissions of agricultural land use in China based on IPCC method and LMDI exponential decomposition Model. Resour. Dev. Mark..

[6] Ma Y.Q., Cao D.G., Luo W.H., Li G.X., Zhang W.F., Li Y.Y. (2024). The evaluation of the livestock and poultry production of carbon emissions in China based on life cycle assessment. China Environ. Sci..

[7] Yang L., Yang Q. (2025). Measurement and zoning of carbon balance in full life cycle of grain production in Gansu section of Yellow River basin. Bull. Soil Water Conserv..

[8] Gao H., Liu T.H., Yang Y.Y., Liu X.R., Yang Y. (2025). Spatiotemporal evolution characteristics of coordination between grain carbon emissions and food security in the Huaihe River Eco- Economic Belt. J. Agric. Resour. Environ..

[9] Gong S., Xu H., Ji Y., Fu Z., Gao M.M., Cheng T., Chen D. (2025). Analysis of spatial and temporal evolution, driving factors and decoupling effects of agricultural carbon emissions in Anhui. Discov. Sustain..

[10] Lin B.Q. (2022). High-quality economic growth in China during the carbon neutrality process. Econ. Res. J..

[11] Zhu Q., Peng X.Z., Lu Z.M., Wu K.Y. (2009). Factor decomposition and empirical analysis of carbon emissions from energy consumption in China. Resour. Sci..

[12] Xu G.Q., Cai Z., Feng S.W. (2021). Spatiotemporal variations and influencing factors of carbon emissions based on a two-stage LMDI model: A case study of Jiangsu Province. Soft Sci..

[13] Chaudhary A., Gustafson D., Mathys A. (2018). Multi-indicator sustainability assessment of global food systems. Nat. Commun..

[14] Song X.Y., Zhou X. (2025). Research on the evaluation of food security in Shandong province based on the entropy-weighted TOPSIS model and analysis of obstacles. Sci. Technol. Econ..

[15] Gong Y.D., Ma Q.Y. (2016). Evaluation of food security in Henan province based on fuzzy matter element model. Shandong Agric. Sci..

[16] Hasegawa T., Sakurai G., Fujimori S. (2021). Extreme climate events increase risk of global food insecurity and adaptation needs. Nat. Food..

[17] Brankovt T., Matkovskib B., Jeremićm M., Đurić I. (2021). Food self-sufficiency of the SEE countries; it is the region prepared for a future crisis. Sustainability.

[18] Song X.Q., Xue J.L., Gao H.X., Li X., Huang W. (2025). Theoretical analysis and modelling of resilience in China’s arable land utilization systems. Acta. Geogr. Sin..

[19] Xu J., Chen G. (2024). Synergistic effects of ecological security and food security in Henan province’s arable land and their influencing factors. J. Saf. Environ..

[20] Yang Y.D., Yao C.S., Liu W.F. (2024). Spatiotemporal characteristics and driving factors of China’s food security system transformation. Acta. Geogr. Sin..

[21] An Y., Tan X.L., Li Y.Q., Zhou Z., Yu H.L., Ren H. (2022). Spatio-temporal evolution characteristics and influencing factors of cultivated land functions in the Dongting Lake Area. Sci. Geogr. Sin..

[22] Soltani A., Alimagham S.M., Nehbandani A., Torabi B., Zeinali E., Zand E., Vadez V., van Loon M.P., van Ittersum M.K. (2020). Future food self-sufficiency in Iran: A model–based analysis. Glob. Food. Sec..

[23] Meng F., Tan Y.Z., Chen H., Xiong W.Y. (2022). Spatiotemporal pattern evolution and influencing factors of cultivated land non-grain conversion in China. China Land Sci..

[24] Wu H.Y., Huang H.J., Cheng W.K., Meng Y. (2022). Estimation and spatiotemporal analysis of the carbon emission efficiency of crop production in China. J. Clean. Prod..

[25] Fan Y.X., He C.F., Wang X.Y., Zhang J.W. (2025). Coupled coordination of population-food-agricultural carbon emission efficiency and agricultural economy in the central Yunnan urban agglomeration and its influencing factors. Environ. Sci..

[26] Wang Y.Y., Cai Y.P., Zhang P. (2025). Decoupling effect and driving factors of net carbon emissions and economic development in the Pearl River Delta urban agglomeration. Acta Ecol. Sin..

[27] Tian Y., Zhang J.B., Li B. (2012). Research on agricultural carbon emissions in China: Measurement, spate-temporal comparison and decoupling effect. Resour. Sci..

[28] He B., Zhou D. (2025). Analysis and performance evaluation of the decoupling relationship between carbon emissions and economic development in China’s agriculture. Pol. J. Environ. Stud..

[29] Jiang Y., Wei C.H., Dong Z.J., Wang Y., Zhan C., Huo Z.Y. (2022). Analysis of time-series characteristics and decoupling elasticity of carbon emissions from grain production in Jiangsu province. Jiangsu Agric. Sci..

[30] Qing X.G., Ai Z.Y. (2007). On regional distribution of rice cultivation in Hunan Province. Agric. Mod. Res..

[31] Zhao X.Y., Zhang T.T., Xie X.T., Peng Y.Z., Zhao Q. (2025). Analysis of the driving factors of carbon emissions and decoupling in the Yangtze River Delta Region. Res. Environ. Sci..

[32] Gong Q.X., Guo G.X. (2024). Decoupling relationship between logistics growth and carbon emissions and driving factors in Chongqing: A novel decomposition framework. J. Environ. Manag..

[33] Bbennetzen E.H., Smith P., Porter J.R. (2016). Decoupling of greenhouse gas emissions from global agricultural production: 1970–2050. Glob. Change Biol..

[34] He Y.Y., Wei Z.X. (2021). The relationship between industrial carbon emissions and economic growth: A validated analysis based on the decoupling between speed and quantity. J. Hunan Norm. Univ..

[35] Qian F.K., Wang X.G., Gu H.L., Wang D.P., Li P.F. (2024). Spatial-temporal differentiation characteristics and key driving factors of agricultural carbon emissions in the three northeastern provinces of China. Chin. J. Eco-Agric..

[36] Li B., Wang C.Y., Zhang J.B. (2019). Dynamic evolution and spatial spillover of China’s agricultural net carbon sink. Chin. J. Popul. Resour..

[37] Xia S.Y., Li J.Z., Zheng Y.J. (2025). Research on the change and prediction of land use carbon emissions in Xiangxi Prefecture under the “dual carbon” goal. Remote Sens. Nat. Resour..

[38] Lei X.P., Qiu G.H. (2016). Empirical study about the carrying capacity evaluation of regional resources and environment based on entropy-weight TOPSIS model. Acta. Sci. Circumst..

[39] Liu S.Y., Li S.Q. (2023). Decoupling elasticity and drivers of agricultural carbon emissions in Hunan province—Based on Tapio decoupling model and LMDI analysis. J. Sichuan Agric. Univ..

[40] Liu H.J. (2022). Research on sustainable utilization of cultivated land in Hunan province based on the ecological footprint model. J. Anhui Agric. Sci..

[41] Deng Y.Y., Gong X.H., He Q.H., Zheng W.W. (2020). Spatiotemporal characteristics and influencing factors of agricultural carbon emissions in Hunan Province. J. Hengyang Norm. Univ..

[42] Batoukhteh F., Darzi-naftchali A. (2024). A global study on decoupling greenhouse gas emissions from agricultural development. Environ. Dev. Sustain..

[43] An Y., Tan X.L., Tan J.Y., Yu H.L., Wang Z.K., Li W.Z. (2021). Evolution of crop planting structure in traditional agricultural areas and its influence factors: A case study in Hunan Province. Econ. Geogr..

[44] Liu X.Z., Li Y. (2023). Relationship between urbanization and carbon emission in the Chang-Zhu-Tan region at the county level. Environ. Sci..

[45] Wang C.J., Wang P., Hong Y.Q., Chen Q.H., Luo J.Y., Xu H.Q. (2025). Regional variations in the carbon footprint of Hunan province’s crop production and analysis of its driving factors. Res. Agric. Mod..

[46] Chowdhury R.B., Moore G.A., Weatherley A.J., Weatherley A.J., Arora M. (2017). Key sustainability challenges for the global phosphorus resource, their implications for global food security, and options for mitigation. J. Clean. Prod..

[47] Reza E. (2025). An integrated approach for three-layer location-allocation in a green supply chain. Int. J. Logist. Syst. Manag..

